# Clinicopathological and molecular predictors of [^18^F]FDG-PET disease detection in HER2-positive early breast cancer: RESPONSE, a substudy of the randomized PHERGain trial

**DOI:** 10.1007/s00259-024-06683-0

**Published:** 2024-04-08

**Authors:** Antonio Llombart-Cussac, Aleix Prat, José Manuel Pérez-García, José Mateos, Tomás Pascual, Santiago Escrivà-de-Romani, Agostina Stradella, Manuel Ruiz-Borrego, Begoña Bermejo de las Heras, Marleen Keyaerts, Patricia Galvan, Fara Brasó-Maristany, Juan José García-Mosquera, Thomas Guiot, María Gion, Miguel Sampayo-Cordero, Serena Di Cosimo, Jhudit Pérez-Escuredo, Manuel Atienza de Frutos, Javier Cortés, Geraldine Gebhart

**Affiliations:** 1 Hospital Arnau de Vilanova, FISABIO, Valencia, Spain; 2 Universidad Católica de Valencia, Valencia, Spain; 3 Medica Scientia Innovation Research (MEDSIR), Barcelona, Spain; 4 Hospital Clínic i Provincial de Barcelona, Barcelona, Spain; 5 University of Barcelona, Barcelona, Spain; 6 Translational Genomics and Targeted Therapies in Solid Tumors Lab., Barcelona, Spain; 7 International Breast Cancer Center, Pangea Oncology, QuironSalud Group, Barcelona, Spain; 8 IEC Barcelona, Barcelona, Spain; 9 Hospital Universitari Vall Hebrón. Vall d’Hebron Institute of Oncology, Barcelona, Spain; 10 ICO L’Hospitalet, Barcelona, Spain; 11 Hospital Virgen del Rocío, Seville, Spain; 12 HCU Valencia, INCLIVA, Universidad de Valencia (CIBERONC-ISCIII, Madrid), Valencia, Spain; 13 Vrije Universiteit Brussel, Brussels, Belgium; 14 Dr. Rosell Oncology Institute (IOR), Dexeus University Hospital, Pangaea Oncology, Quironsalud Group, Barcelona, Spain; 15 Université Libre de Bruxelles, Hôpital Universitaire de Bruxelles, Institute Jules Bordet, Brussels, Belgium; 16 Hospital Ramón y Cajal, Madrid, Spain; 17 Fondazione IRCCS Istituto Nazionale Dei Tumori, Milano, Italy; 18 Universidad Europea de Madrid, Faculty of Biomedical and Health Sciences, Department of Medicine, Madrid, Spain

**Keywords:** Disease detection, HER2-positive, Early breast cancer, [^18^F]FDG-PET, Predictors

## Abstract

**Background:**

The PHERGain study (NCT03161353) is assessing early metabolic responses to neoadjuvant treatment with trastuzumab-pertuzumab and chemotherapy de-escalation using a [^18^Fluorine]fluorodeoxyglucose-positron emission tomography ([^18^F]FDG-PET) and a pathological complete response-adapted strategy in HER2-positive (HER2+) early breast cancer (EBC). Herein, we present RESPONSE, a PHERGain substudy, where clinicopathological and molecular predictors of [^18^F]FDG-PET disease detection were evaluated.

**Methods:**

A total of 500 patients with HER2 + EBC screened in the PHERGain trial with a tumor size > 1.5 cm by magnetic resonance imaging (MRI) were included in the RESPONSE substudy. PET[−] criteria entailed the absence of  ≥ 1 breast lesion with maximum standardized uptake value (SUVmax) ≥ 1.5 × SUVmean liver + 2 standard deviation. Among 75 PET[−] patients screened, 21 with SUVmax levels < 2.5 were randomly selected and matched with 21 PET[+] patients with SUVmax levels ≥ 2.5 based on patient characteristics associated with [^18^F]FDG-PET status. The association between baseline SUVmax and [^18^F]FDG-PET status ([−] or [+]) with clinicopathological characteristics was assessed. In addition, evaluation of stromal tumor-infiltrating lymphocytes (sTILs) and gene expression analysis using PAM50 and Vantage 3D™ Cancer Metabolism Panel were specifically compared in a matched cohort of excluded and enrolled patients based on the [^18^F]FDG-PET eligibility criteria.

**Results:**

Median SUVmax at baseline was 7.2 (range, 1–39.3). Among all analyzed patients, a higher SUVmax was associated with a higher tumor stage, larger tumor size, lymph node involvement, hormone receptor-negative status, higher HER2 protein expression, increased Ki67 proliferation index, and higher histological grade (*p* < 0.05). [^18^F]FDG-PET [−] criteria patients had smaller tumor size (*p* = 0.014) along with the absence of lymph node involvement and lower histological grade than [^18^F]FDG-PET [+] patients (*p* < 0.01). Although no difference in the levels of sTILs was found among 42 matched [^18^F]FDG-PET [−]/[+] criteria patients (*p* = 0.73), [^18^F]FDG-PET [−] criteria patients showed a decreased risk of recurrence (ROR) and a lower proportion of PAM50 HER2-enriched subtype than [^18^F]FDG-PET[+] patients (*p* < 0.05). Differences in the expression of genes involved in cancer metabolism were observed between [^18^F]FDG-PET [−] and [^18^F]FDG-PET[+] criteria patients.

**Conclusions:**

These results highlight the clinical, biological, and metabolic heterogeneity of HER2+ breast cancer, which may facilitate the selection of HER2+ EBC patients likely to benefit from [^18^F]FDG-PET imaging as a tool to guide therapy.

**Trial registration:**

Clinicaltrials.gov; NCT03161353; registration date: May 15, 2017.

**Supplementary Information:**

The online version contains supplementary material available at 10.1007/s00259-024-06683-0.

## Introduction

Human epidermal growth factor receptor 2-positive (HER2+) breast cancer (BC) is a clinically and biologically heterogeneous disease [1] characterized by the amplification of the *ERBB2/HER2* gene and/or overexpression of its related kinase receptor protein [2]. This tumor subtype comprises around 15–20% of all BCs and has been associated with a high risk of recurrence and poor outcomes [3, 4].

HER2-targeted agents have radically improved the prognosis of HER2+ early BC (EBC) offering the possibility to de-escalate standard chemotherapy in selected subgroups [5]. The neoadjuvant setting provides the best scenario for treatment de-escalation considering that patients achieving a pathological complete response (pCR) have favorable long-term outcomes in terms of disease-free survival and overall survival [6].

Several studies have investigated predictive factors of pCR to neoadjuvant treatment. Imaging tools that could guide the response to preoperative therapy are of great interest, mainly the potential utility of [^18^Fluorine]fluorodeoxyglucose ([^18^F]FDG)-positron emission tomography (PET). The association between early treatment response on ([^18^F]FDG-PET) and clinical outcomes has been evaluated in patients with HER2+ BC in metastatic and neoadjuvant settings [4, 7–11]. These studies demonstrated that early metabolic evaluation using [^18^F]FDG-PET might identify HER2+ tumors with high anti-HER2 sensitivity and an increased likelihood of achieving a pCR on neoadjuvant HER2 blockade [10–12].

PHERGain (NCT03161353) is an international, randomized, open-label, phase II trial that aims to assess the efficacy of a chemotherapy-free strategy based on a dual HER2 blockade with trastuzumab-pertuzumab (+ endocrine therapy for hormone receptor [HR]-positive tumors) as neoadjuvant and adjuvant therapy in HER2+ EBC patients through an [^18^F]FDG-PET and pCR-adapted strategy [5]. It is assessing whether [^18^F]FDG-PET along with the pathological response could identify tumors with high anti-HER2 sensitivity to avoid standard chemotherapy in subsequent cycles (neoadjuvant and adjuvant setting) in patients with an early [^18^F]FDG-PET response that achieved a pCR with exclusive dual HER2 blockade with trastuzumab-pertuzumab. For this purpose, breast lesions must be SUVmax ≥ 1.5 × SUVmean liver + 2 SD, an inclusion criterion that was not met in a significant number of patients (screening failure).

The clinical, pathological, and molecular characteristics of these screening failures need to be investigated in order to guide more adequate patient selection. Numerous studies have reported an association between tumor [^18^F]FDG uptake in [^18^F]FDG-PET and both molecular subtypes of BC, as well as clinicopathological characteristics [11, 13–22].

To investigate this, we designed the RESPONSE, a substudy of the PHERGain trial, in which we aimed to obtain information regarding the clinicopathological and molecular characteristics of tumors that can impact on the [^18^F]FDG-PET evaluation of HER2+ tumors, and, therefore, on its ability of prediction (diagnosis/response). Here, we report the results of this subanalysis, which included all patients with HER2+ EBC screened in the PHERGain trial.

## Material and methods

See the full version of the “Material and methods” section as supplementary material (references [5, 23–25]).

Our substudy included patients from the PHERGain trial (NCT03161353) [5] that had previously been untreated, HER2+ , stage I-IIIA, invasive and operable BC, with ≥ 1 [^18^F]FDG-PET target breast lesions ≥ 1.5 cm by MRI or ultrasound at baseline. PET[+] criteria patients were those with SUVmax levels ≥ 2.5 based on the formula for breast lesions (SUVmax ≥ 1.5 × SUVmean liver + 2 standard deviation [SD]), and their matched PET[−] counterparts had SUVmax levels ≥ 2.5. A visual comparison of two typical patients of each cohort is shown in Supplementary Fig. 1.

The objectives of this substudy were as follows: (1) to evaluate the association between SUVmax and PET[+] and PET[−] criteria at baseline with the clinicopathological features in all screened patients with a tumor size > 1.5 cm by MRI (*n* = 500) and (2) to analyze differences in sTILs and gene expression using PAM50 (intrinsic subtyping and ROR scores) and Vantage 3D™ Cancer Metabolism Panel in a matched cohort of 21 PET[−] and 21 PET[+] patients.

This study was performed in accordance with guidelines of the International Conference on Harmonization and ethical principles outlined in the Declaration of Helsinki. Written informed consent was required before enrolment, and all participants agreed to study-specific procedures. Approvals from the following regulatory authorities and ethics committees were obtained: Comité Ético de la Investigación con Medicamentos del Hospital Arnau de Vilanova (Spain), Comité de Protection des Personnes EST-III Hôpital de Brabois (France), Ethikkommission der Medizinischen Fakultät Heidelberg (Germany), The Ethics Committee of the Institut Jules Bordet (Belgium), CEIC—Comissão de Etica para a Investigação Clinica, Parque Saude Lisboa (Portugal), Fulham Research Ethics Committee Charing Cross Hospital (United Kingdom), and Comitato Etico ASL Brindisi, Comitato Etico della provincia Monza Brianza, Comitato Etico Istituto Europeo di Oncologia e Centro Cardiologico Monzino, Comitato Etico Val Padana, Comitato Etico di Area Vasta Emilia Centro, and Comitato Etico dell’Area Vasta Emilia Nord (Italy).

### Statistical analysis

In all screened patients of PHERGain trial with a tumor size > 1.5 cm by MRI, unadjusted and adjusted analyses were performed to assess the relationship between SUVmax at baseline and [^18^F]FDG-PET status ([−] or [+]) with clinicopathological characteristics (age, tumor stage, tumor size, nodal status, HR status, HER2 immunohistochemistry status, carcinoma type, tumor grade, and Ki67 proliferation index).

Subsequently, we randomly selected 21 PET [−] criteria patients with SUVmax levels < 2.5 among 75 PET [−] criteria patients screened. We matched them with 21 patients with SUVmax levels ≥ 2.5 (PET[+] patients) based on patient characteristics previously associated with [^18^F]FDG-PET status (tumor size, nodal involvement, and histological grade). PET[−] and PET[+] patients had the same tumor stage, nodal status, and histological grade in the matched analysis. PAM50 intrinsic subtyping, ROR scores, and cancer metabolism gene expression were compared according to [^18^F]FDG-PET status in the matched cohorts.

For all statistical analyses, *p*-value < 0.05 was considered statistically significant. Multiple testing issues with gene expression were controlled with a false discovery rate using a threshold of *q*-value < 5%. Results from overall correlative analyses should be considered descriptive because of the small number of samples and unadjusted sequential testing.

## Results

### Patients

**Fig. 1:**
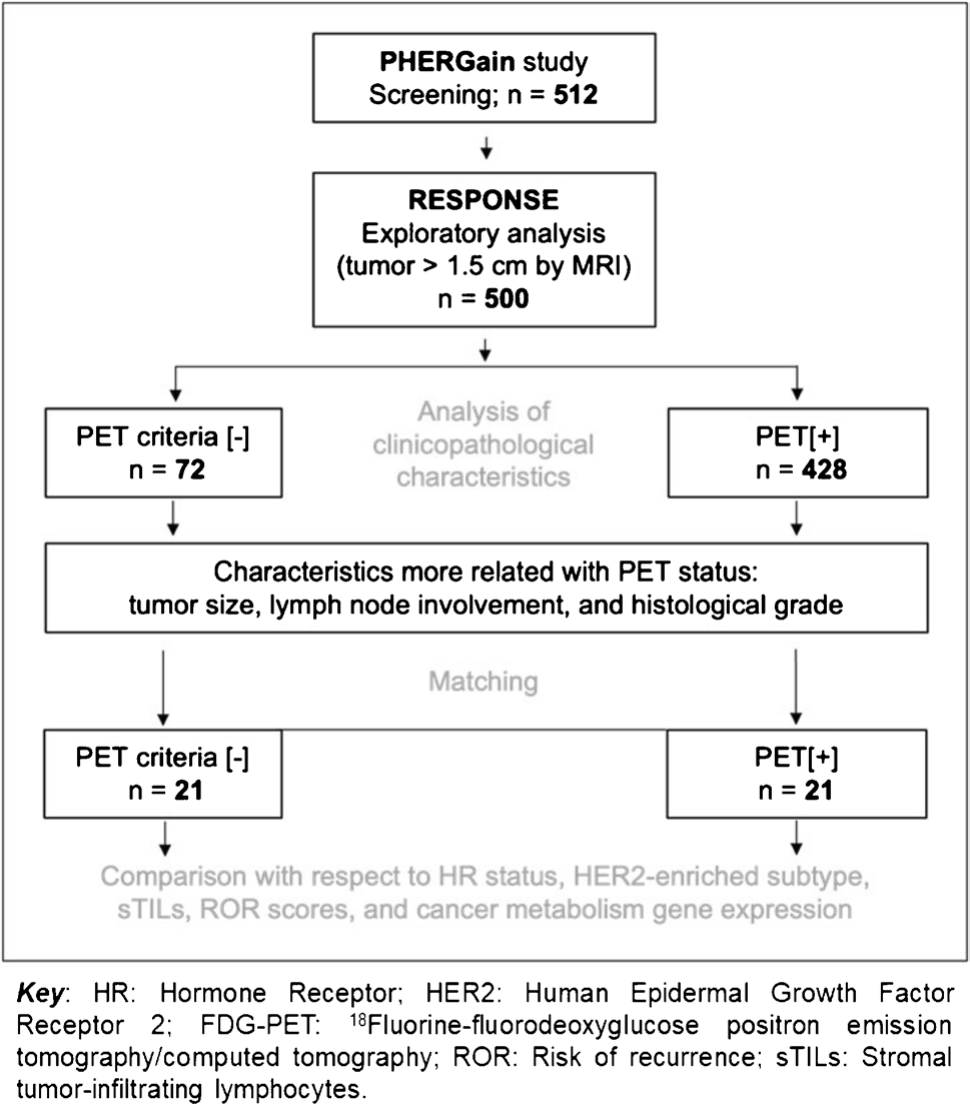
Patient disposition

All patients screened in the PHERGain trial with a tumor size > 1.5 cm by MRI were included in the RESPONSE substudy, resulting in a total of 500 patients. Extensive biomarker analysis (sTILs, ROR scores and molecular subtyping by PAM50, and cancer metabolism gene expression) was specifically performed in a matched cohort of 21 PET [−] and 21 PET [+] criteria patients. Figure 1 shows the patient disposition.

Among patients in this substudy, the median age was 52 years (range, 20–83), 42.2% (211/500) had node-positive disease, 68.2% (341/500) had HR-positive tumors, and 75.2% (376/500) had HER2 3+ tumors by immunohistochemistry. Median SUVmax at baseline was 7.2 (range, 1–39.3), and median tumor size by MRI was 33 mm (range, 15.3–157) (Table 1).

**Table 1:** Clinicopathological characteristics (analysis of the entire population and by [^18^F]FDG-PET status)

Characteristics	All patients (*N* = 500)	PET [−] criteria (*N* = 72)	PET[+] criteria (*N* = 428)	Unadjusted OR (95% CI), *p*-value	Adjusted OR (95% CI), *p*-value
Age in years, median (range)	52 (20–83)	52 (36–83)	51 (20–82)	1 (0.99–1.04), *p* = 0.134	1 (0.99–1.04), *p* = 0.170
Tumor size by MRI in millimeters, median (range)	33 (15.3–157)	32 (16–100)	33 (15.3–157)	0.99 (0.98–1), *p* = 0.231	1 (0.99–1.01) *p* = 0.982
Tumor stage; *n* (%)				5.2 (2.9–9.0)	3.0 (1.6–5.5)
I	75 (15.0)	28 (38.9)	47 (11.0)	*p* < 0.001	*p* = 0.014
II	355 (71.0)	40 (55.6)	315 (73.6)	-	-
IIIA	70 (14.0)	4 (5.6)	66 (15.4)	-	-
Tumor size (T); *n* (%)				2.2 (1.1–4.1)	2.0 (1.0–3.9)
T1	65 (13.0)	16 (22.2)	49 (11.4)	*p* = 0.014	*p* = 0.036
T2	353 (70.6)	46 (63.9)	307 (71.7)	-	-
T3	82 (16.4)	10 (13.9)	72 (16.8)	-	-
SUVmax at baseline, median (range)	7.2 (1–39.3)	2.7 (1–4.41)	8.0 (2.1–39.3)	0.18 (0.11–0.26), *p* < 0.01	0.17 (0.1–0.25), *p* < 0.01
Nodal status (N); *n* (%)				4.9 (2.6–10)	4.2 (2.2–8.7)
N0	289 (57.8)	61 (84.7)	228 (53.3)	*p* < 0.01	*p* < 0.01
N1	211 (42.2)	11 (15.3)	200 (46.7)	**-**	**-**
HR status; *n* (%)				1.2 (0.7–2)	0.8 (0.5–1.6)
[ −]	159 (31.8)	21 (29.2)	138 (32.2)	*p* = 0.604	*p* = 0.651
[ +]	341 (68.2)	51 (70.8)	290 (67.8)	-	-
HER2 IHC status; *n* (%)				0.7 (0.4–1.1)	0.87 (0.5–1.6)
2 +	124 (24.8)	23 (31.9)	101 (23.6)	*p* = 0.131	*p* = 0.641
3 +	376 (75.2)	49 (68.1)	327 (76.4)	-	-
Ductal carcinoma, *n* (%)				0.46 (0.26–0.82)	0.57 (0.31–1.1)
No	89 (17.8)	21 (29.2)	68 (15.9)	*p* < 0.01	*p* = 0.069
Yes	411 (82.2)	51 (70.8)	360 (84.1)	-	-
Tumor grade; *n* (%)				*p* < 0.01	*p* < 0.01
G1-2	208 (41.6)	40 (55.6)	168 (39.3)	**-**	**-**
G3	199 (39.8)	13 (18.1)	186 (43.5)	0.29 (0.15–0.55)	0.34 (0.17–0.65)
Gx	93 (18.6)	19 (26.4)	74 (17.3)	1.08 (0.58–1.97)	1.17 (0.60–2.21)
Ki67 proliferation index; *n* (%)				0.69 (0.38–1.3)	0.89 (0.47–1.8)
≤ 20	81 (16.2)	15 (20.8)	66 (15.4)	*p* = 0.251	*p* = 0.739
> 20	419 (83.8)	57 (79.2)	362 (84.6)	-	-

Percentages may not total 100% due to rounding

*G* grade, *HER* human epidermal growth factor receptor, *HR* hormone receptor, *IHC* immunohistochemistry, *MRI* magnetic resonance imaging, *SUVmax* maximum standardized uptake value

### Correlation between SUVmax at baseline and clinicopathological characteristics in all patients

**Fig. 2:**
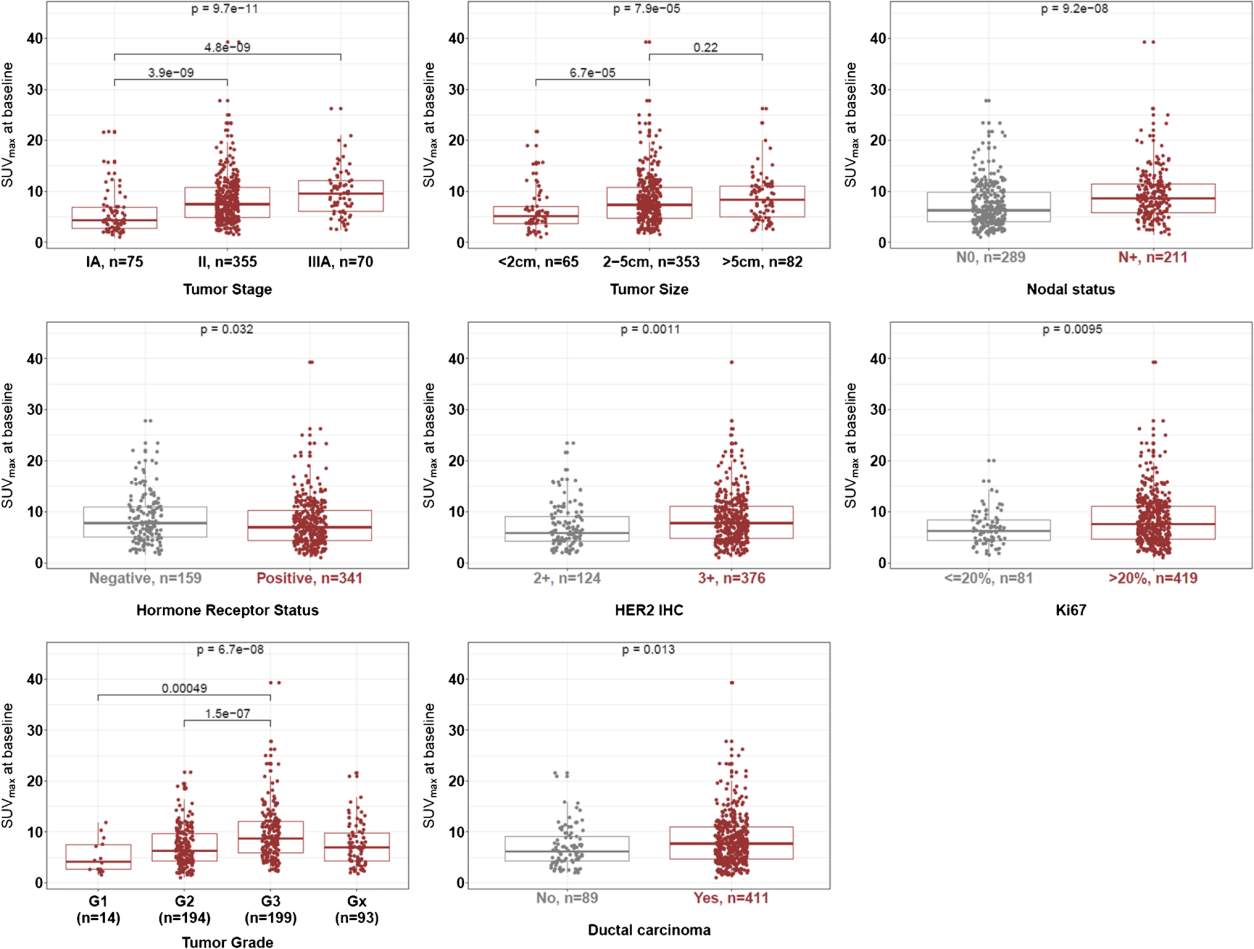
Association between SUVmax at baseline and clinicopathological characteristics

SUVmax at baseline was higher in tumors with stage IIIA (*p* < 0.01), a diameter ≥ 2 cm (*p* < 0.01), lymph node involvement (*p* < 0.01), HR-negative status (*p* = 0.032), higher HER2 protein expression (*p* < 0.01), increased Ki67 proliferation index (*p* = 0.01), higher histological grade (*p* < 0.01), and ductal carcinoma type (*p* = 0.013) (Fig. 2).

### Association between [^18^F]FDG-PET status and clinicopathological characteristics in all patients

Median SUVmax at baseline was 2.7 (range, 1.0–4.41) and 8.0 (range, 2.1–39.3) in PET[−] criteria (screening failures due to the lack of ≥ 1 breast lesion evaluable by [^18^F]FDG-PET) and PET [+] criteria patients (included) (*p* < 0.01), respectively. Median tumor size by MRI was 32 mm (range, 16–100) and 33 mm (range, 15.3–157) in PET [−] and PET [+] criteria patients (*p* = 0.231), respectively (Table 1).

In an unadjusted analysis, PET [−] criteria patients showed more early-stage tumors (*p* < 0.001), decreased tumor size (*p* = 0.014), absence of lymph node involvement (*p* < 0.01), more non-ductal histology (*p* = 0.013), and lower histological grade (*p* < 0.01) (Table 1).

Using an adjusted analysis, we selected patient’s characteristics more likely to predict [^18^F]FDG-PET status to match PET [−] and PET[+] criteria patients. PET [−] criteria patients had a lower tumor stage (odds ratio (OR) 3.0, 95% CI 1.1–4.1; *p* = 0.014), smaller tumor size (OR 2.2, 95% CI 1.1–4.1; *p* = 0.014), absence of nodal involvement (OR 4.2, 95% CI 2.2–8.7; *p* < 0.01), and lower histological grade (OR 0.32, 95% CI 0.16–0.6; *p* < 0.01) (Table 1).

### sTILs, PAM50 intrinsic subtyping and ROR scores, and cancer metabolism gene expression according to [^18^F]FDG-PET status in matched cohorts

After matching for tumor size, lymph node involvement, and histological grade, differences in sTILs and gene expression by PAM50 (intrinsic subtyping and ROR scores) and Vantage 3D™ Cancer Metabolism Panel were analyzed in a cohort of 21 PET[−] and 21 PET[+] criteria patients (Table 2 and Fig. 1).

**Table 2:** Clinicopathological and biological characteristics for matched samples by [^18^F]FDG-PET status

Characteristics	PET [−] criteria (*N* = 21)	PET [+] criteria (*N* = 21)	*p*-value
Age in years, median (range)	54 (40–65)	53 (30–77)	0.865
Tumor size (T); *n* (%)			
T1	9 (42.9)	9 (42.9)	1
T2	11 (52.4)	11 (52.4)	-
T3	1 (4.8)	1 (4.8)	-
SUV_max_ at baseline, median (range)	2.1 (1–2.5)	9.2 (5.6–21.7)	< 0.001
Nodal status (N); *n* (%)			
N0	15 (71.4)	15 (71.4)	1
N1	6 (28.6)	6 (28.6)	-
Tumor grade, *n* (%)			
G1	1 (4.8)	1 (4.8)	1
G2	14 (66.7)	14 (66.7)	-
G3	5 (23.8)	5 (23.8)	-
Gx	1 (4.8)	1 (4.8)	-
Ductal carcinoma, *n* (%)			
No	2 (9.5)	2 (9.5)	1
Yes	19 (90.5)	19 (90.5)	-
HR status; *n* (%)			
[ −]	8 (38.1)	13 (61.9)	0.182
[ +]	13 (61.9)	8 (38.1)	-
HER2 IHC status; *n* (%)			
2 +	7 (33.3)	4 (19)	0.45
3 +	14 (66.7)	17 (81)	-
ROR (subtype only); *n* (%)			
Low	5 (23.8)	1 (4.8)	0.289
Medium	3 (14.3)	3 (14.3)	-
High	13 (61.9)	17 (81)	-
ROR (subtype + proliferation); *n* (%)			
Low	4 (19)	2 (9.5)	1
Medium	5 (23.8)	7 (33.3)	-
High	12 (57.1)	12 (57.1)	-
Ki67 proliferation index; *n* (%)			
≤ 20	13 (61.9)	14 (66.7)	1
> 20	8 (38.1)	7 (33.3)	-

Percentages may not total 100% due to rounding

*G* grade, *HER* human epidermal growth factor receptor, *HR* hormone receptor, *IHC* immunohistochemistry, *ROR* risk of recurrence, *SUVmax* maximum standardized uptake value

**Fig. 3:**
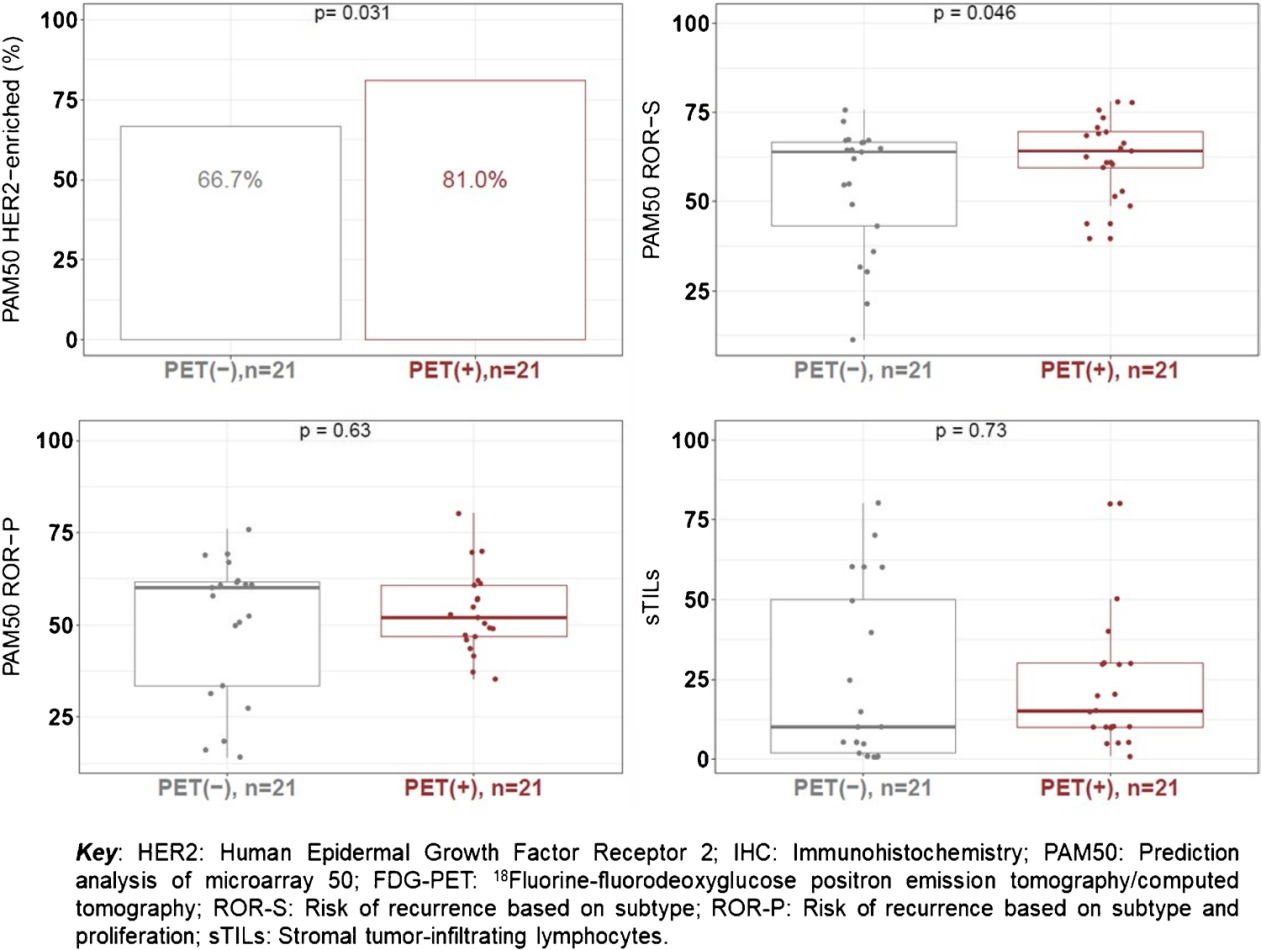
Stromal tumor-infiltrating lymphocytes, HER2-enriched subtype, and risk of recurrence scores according to [^18^F]FDG-PET status (− / +)

No differences in the level of sTILs was found among matched PET [−] and [+] criteria patients with a median score of 10% and 15% in PET [−] and PET[+] criteria patients (*p* = 0.73), respectively (Fig. 3). The percentages of patients in the different levels of sTILs were the same regardless of the [^18^F]FDG-PET status ([−] or [+]): 66.7% (14/21) of the patients had low sTILs (sTILs < 30%), 28.6% (6/21) intermediate sTILs (sTILs 30–75%), and 4.8% (1/21) high sTILs (sTILs ≥ 75%).

PET [−] criteria patients had slightly lower ROR-S scores than PET[+] criteria patients (median, 63.9 (range 11.3–75.7) vs. 64.2 (range, 39.7–78)) and a lower proportion of HER2-enriched subtype (66.7% (14/21 PET [−] criteria) vs. 81.0% (17/21 PET[+] criteria) by PAM50 (*p* < 0.05). No significant differences for ROR-P scores were observed according to [^18^F]FDG-PET status (*p* = 0.63) (Fig. 3).

**Fig. 4:**
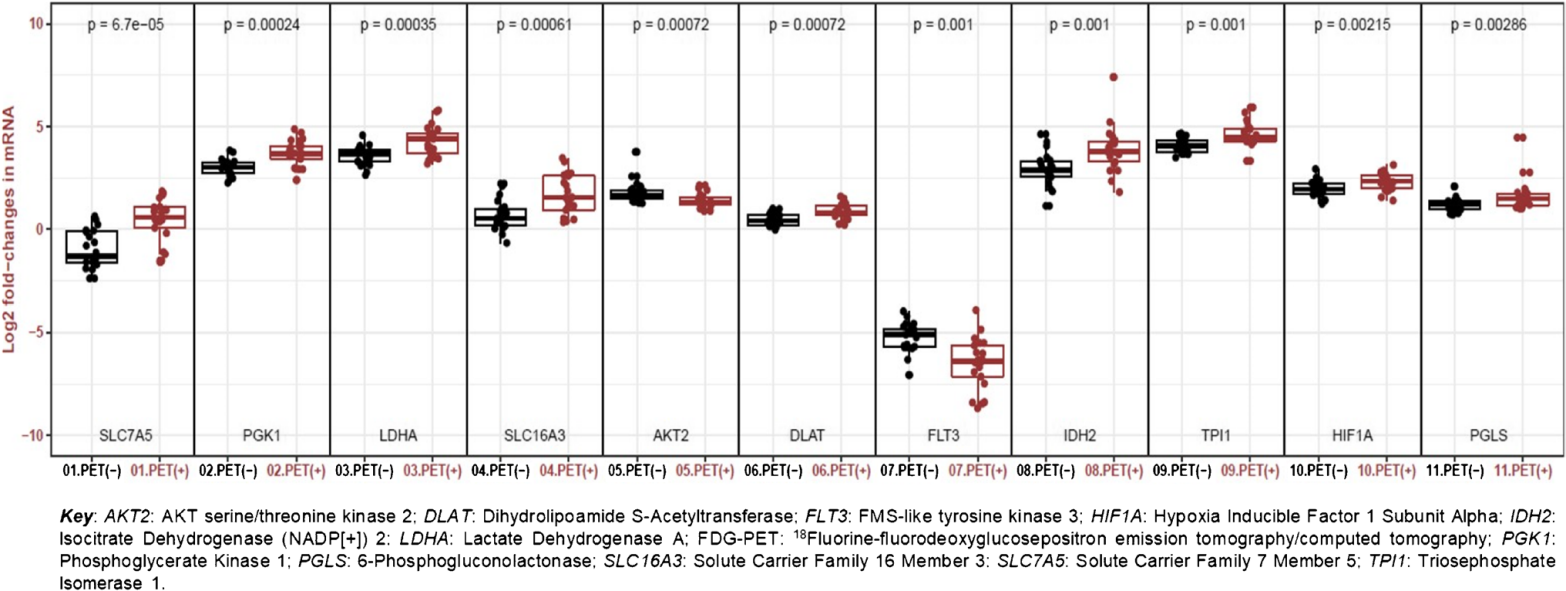
Box plot of cancer metabolism gene expression according to [^18^F]FDG-PET status (− / +)

Genes involved in glucose metabolism (*DLAT*, *IDH2*, *LDHA*, *PGK1*, *PGLS*, and *TPI1*), hypoxia signaling (*HIF1A*), and carbon metabolism (*SLC7A5* and *SLC16A3*) were under-expressed in PET [−] criteria patients, whereas genes involved in the mTOR pathway (*AKT2*) and growth factor receptor (*FLT3*) were overexpressed compared to PET[+] patients (false discovery rate *q* < 0.05) (Fig. 4).

## Discussion

The PHERGain trial [5] only included patients whose breast tumors were ≥ 1.5 cm in diameter (MRI or ultrasound) in order to reduce screening failures due to the absence of evaluable breast lesions by [^18^F]FDG-PET. However, despite this inclusion criterion and the aggressive behavior of HER2+ tumors, around 15% of the patients were excluded as a result of the lack of  ≥ 1 breast lesions evaluable by [^18^F]-FDG-PET [26].

[^18^F]FDG-PET response after two cycles of treatment was critical for treatment decision-making in patients included in the group of the PHERGain trial not receiving chemotherapy [5]. The adaptive design of this trial, therefore, made it necessary to select patients with breast lesions identified by [^18^F]FDG-PET, defined as SUVmax ≥ 1.5 × SUVmean liver + 2 SD. This strict requirement is the main factor responsible for this significant rate of screening failures. For this reason, a better understanding of clinical and molecular determinants of [^18^F]FDG-PET disease detection could help to better select patients for studies that use [^18^F]FDG-PET as assessment method.

The median SUVmax at baseline in our entire analyzed population was 7.2 (range 1–39.3), which is similar to previous studies including patients with HER2+ tumors [19, 27]. Additionally, our findings are in line with previous studies in patients with EBC that have demonstrated a relationship between SUVmax and several classical clinicopathological characteristics such as clinical stage [14], HR status [15, 16], Ki67 proliferation index [11, 17], tumor size [18], and histological grade [19–21] regardless of BC subtype. Nevertheless, our results are of particular interest because they were specifically generated in patients with HER2+ tumors.

One of the main objectives of this substudy was the analysis of the differences in sTILs and gene expression by PAM50 (intrinsic subtyping and ROR scores) and Vantage 3D™ Cancer Metabolism Panel in a matched cohort of excluded and enrolled patients in the PHERGain trial based on the [^18^F]FDG-PET inclusion criteria. TILs are predictive biomarkers of response to neoadjuvant therapy in patients with HER2+ tumors [28, 29]. Interestingly, we did not find differences in the levels of sTILs among matched PET [−]/[ +] criteria patients.

Compared with PET[+] criteria patients, PET [−] criteria patients had lower ROR scores, a prognostic factor that has been considered superior to other classical clinicopathological characteristics [30]. Lower ROR scores are associated with a reduced risk of BC relapse in patients with HR+/HER2- EBC [30]. However, the prognostic role of ROR scores in HER2+ tumors remains undetermined.

Our results also showed a higher proportion of PAM50 HER2-enriched tumors and higher levels of HER2-protein expression by immunohistochemistry in PET[+] criteria patients. HER2-enriched subtype and HER2 3+ tumors by immunohistochemistry appeared to be associated with higher pCR rates following anti-HER2-based regimes. These findings are consistent with the capacity of [^18^F]FDG-PET to predict a pCR to neoadjuvant treatment with HER2-targeted therapies [31].

Regarding the gene under-expression we observed in PET[−] criteria patients, with low [^18^F]FDG avidity, the lower expression of *HIF1A* was in concordance with the higher SUVmax detected in patients with BC with high *HIF-1A* expression [32]. On the other hand, the reduction of glucose metabolism, suggested by the lower expression of genes involved in glucose metabolism, could be justified with the fact that tumor cells can switch their metabolic pathway from glucose to other nutrients such as glutamine [33, 34].

Our substudy has five main limitations worth noting. First, its exploratory nature; the results reported here should be interpreted with caution and considered hypotheses-generating. Second, there was a small sample size in the matched cohorts of PET [−] and PET[+] criteria patients (21 patients each); analyses with a larger sample size would have provided more consistent results. Third, we have tested many variables in a small population and the use of a false discovery rate only partially addresses this limitation. Fourth, matching could decrease external validity because the controls become more similar to the cases than expected in the target population. Fifth, due to the high partial volume effect of [^18^F]FDG-PET, the SUV of tumor lesions < 2 cm may be artificially reduced.

## Conclusions

Our findings highlight the clinical, biological, and metabolic heterogeneity of HER2+ breast cancer, which may facilitate to select HER2+ EBC patients likely to benefit from [^18^F]FDG-PET imaging as a tool to guide therapy.

### Supplementary Information

Below is the link to the electronic supplementary material.

Supplementary file1 (DOCX 291 KB)

## Data Availability

Data collected within the RESPONSE substudy will be made available to researchers whose full proposal for their use of the data has been approved by the PHERGain Trial Management Group that includes a qualified statistician. The data required for the approved, specified purposes and the trial protocol will be provided, after completion of a data sharing agreement, that will be set up by the study sponsor. *All data provided are anonymized to respect the privacy of patients who have participated in the trial in line with applicable laws and regulations.* Please address requests for data to the corresponding authors.

## References

[1] Hassett MJ, Li H, Burstein HJ, Punglia RS (2020). Neoadjuvant treatment strategies for HER2-positive breast cancer: cost-effectiveness and quality of life outcomes. Breast Cancer Res Treat.

[2] Schettini F, Prat A (2021). Dissecting the biological heterogeneity of HER2-positive breast cancer. Breast.

[3] González-Santiago S, Saura C, Ciruelos E (2020). Real-world effectiveness of dual HER2 blockade with pertuzumab and trastuzumab for neoadjuvant treatment of HER2-positive early breast cancer (The NEOPETRA Study). Breast Cancer Res Treat.

[4] Lee MI, Jung YJ, Kim DI (2021). Prognostic value of SUVmax in breast cancer and comparative analyses of molecular subtypes: a systematic review and meta-analysis. Medicine (Baltimore).

[5] Pérez-García JM, Gebhart G, Ruiz Borrego M (2021). Chemotherapy de-escalation using an 18F-FDG-PET-based pathological response-adapted strategy in patients with HER2-positive early breast cancer (PHERGain): a multicentre, randomised, open-label, non-comparative, phase 2 trial. Lancet Oncol.

[6] Cortazar P, Zhang L, Untch M (2014). Pathological complete response and long-term clinical benefit in breast cancer: the CTNeoBC pooled analysis. Lancet.

[7] Antunovic L, De Sanctis R, Cozzi L (2019). PET/CT radiomics in breast cancer: promising tool for prediction of pathological response to neoadjuvant chemotherapy. Eur J Nucl Med Mol Imaging.

[8] Ming Y, Wu N, Qian T (2020). Progress and future trends in PET/CT and PET/MRI molecular imaging approaches for breast cancer. Front Oncol.

[9] Liu Q, Wang C, Li P (2016). The role of (18)F-FDG PET/CT and MRI in assessing pathological complete response to neoadjuvant chemotherapy in patients with breast cancer: a systematic review and meta-analysis. Biomed Res Int.

[10] Spring LM, Fell G, Arfe A (2020). Pathologic complete response after neoadjuvant chemotherapy and impact on breast cancer recurrence and survival: a comprehensive meta-analysis. Clin Cancer Res.

[11] Zhao F, Huo X, Wang M (2021). Comparing biomarkers for predicting pathological responses to neoadjuvant therapy in HER2-positive breast cancer: a systematic review and meta-analysis. Front Oncol.

[12] Coudert B, Pierga J-Y, Mouret-Reynier M-A (2014). Use of [(18)F]-FDG PET to predict response to neoadjuvant trastuzumab and docetaxel in patients with HER2-positive breast cancer, and addition of bevacizumab to neoadjuvant trastuzumab and docetaxel in [(18)F]-FDG PET-predicted non-responders (AVATAXHER): an open-label, randomised phase 2 trial. Lancet Oncol.

[13] Murakami W, Tozaki M, Sasaki M (2020). Correlation between 18F-FDG uptake on PET/MRI and the level of tumor-infiltrating lymphocytes (TILs) in triple-negative and HER2-positive breast cancer. Eur J Radiol.

[14] Mori M, Fujioka T, Kubota K (2021). Relationship between prognostic stage in breast cancer and fluorine-18 fluorodeoxyglucose positron emission tomography/computed tomography. J Clin Med.

[15] Arslan E, Çermik TF, Trabulus FDC (2018). Role of 18F-FDG PET/CT in evaluating molecular subtypes and clinicopathological features of primary breast cancer. Nucl Med Commun.

[16] Groheux D, Cochet A, Humbert O (2016). ^18^F-FDG PET/CT for staging and restaging of breast cancer. J Nucl Med.

[17] Surov A, Meyer HJ, Wienke A (2019). Associations between PET parameters and expression of Ki-67 in breast cancer. Transl Oncol.

[18] AbdElaal A, Zaher AM, Abdelgawad MI (2021). Correlation of primary tumor metabolic parameters with clinical, histopathological and molecular characteristics in breast cancer patients at pre-operative staging FDG-PET/CT study. Egypt J Radiol Nucl Med.

[19] Groheux D, Giacchetti S, Hatt M (2013). HER2-overexpressing breast cancer: FDG uptake after two cycles of chemotherapy predicts the outcome of neoadjuvant treatment. Br J Cancer.

[20] Groheux D, Hindie E (2021). Breast cancer: initial workup and staging with FDG PET/CT. Clin Transl Imaging.

[21] Önner H, Canaz F, Dinçer M (2019). Which of the fluorine-18 fluorodeoxyglucose positron emission tomography/computerized tomography parameters are better associated with prognostic factors in breast cancer?. Medicine (Baltimore).

[22] Groheux D, Giacchetti S, Moretti J-L (2011). Correlation of high 18F-FDG uptake to clinical, pathological and biological prognostic factors in breast cancer. Eur J Nucl Med Mol Imaging.

[23] de Azambuja E, Holmes AP, Piccart-Gebhart M (2014). Lapatinib with trastuzumab for HER2-positive early breast cancer (NeoALTTO): survival outcomes of a randomised, open-label, multicentre, phase 3 trial and their association with pathological complete response. Lancet Oncol.

[24] Kos Z, Roblin E, Kim RS (2020). Pitfalls in assessing stromal tumor infiltrating lymphocytes (sTILs) in breast cancer. NPJ Breast Cancer.

[25] Llombart-Cussac A, Cortés J, Paré L (2017). HER2-enriched subtype as a predictor of pathological complete response following trastuzumab and lapatinib without chemotherapy in early-stage HER2-positive breast cancer (PAMELA): an open-label, single-group, multicentre, phase 2 trial. Lancet Oncol.

[26] Jh O, Lodge MA, Wahl RL (2016). Practical PERCIST: a simplified guide to PET response criteria in solid tumors 1.0. Radiology.

[27] Kitajima K, Miyoshi Y, Sekine T (2021). Harmonized pretreatment quantitative volume-based FDG-PET/CT parameters for prognosis of stage I-III breast cancer: multicenter study. Oncotarget.

[28] Hwang HW, Jung H, Hyeon J (2019). A nomogram to predict pathologic complete response (pCR) and the value of tumor-infiltrating lymphocytes (TILs) for prediction of response to neoadjuvant chemotherapy (NAC) in breast cancer patients. Breast Cancer Res Treat.

[29] Schettini F, Pascual T, Conte B (2020). HER2-enriched subtype and pathological complete response in HER2-positive breast cancer: a systematic review and meta-analysis. Cancer Treat Rev.

[30] Ohnstad HO, Borgen E, Falk RS (2017). Prognostic value of PAM50 and risk of recurrence score in patients with early-stage breast cancer with long-term follow-up. Breast Cancer Res.

[31] Edmonds CE, O’Brien SR, Mankoff DA, Pantel AR (2022). Novel applications of molecular imaging to guide breast cancer therapy. Cancer Imaging.

[32] Jeong Y-J, Jung J-W, Cho Y-Y (2017). Correlation of hypoxia inducible transcription factor in breast cancer and SUVmax of F-18 FDG PET/CT. Nucl Med Rev Cent East Eur.

[33] Robey IF, Stephen RM, Brown KS (2008). Regulation of the Warburg effect in early-passage breast cancer cells. Neoplasia.

[34] El Ansari R, McIntyre A, Craze ML (2018). Altered glutamine metabolism in breast cancer; subtype dependencies and alternative adaptations. Histopathology.

