# Supplementary material for: Clinicopathological and molecular predictors of [18F]FDG-PET disease detection in HER2-positive early breast cancer: RESPONSE, a substudy of the randomized PHERGain trial
Source: Eur J Nucl Med Mol Imaging. 2024 Apr 8;51(9):2733–43. doi: 10.1007/s00259-024-06683-0 (PMC11224085; doi:10.1007/s00259-024-06683-0)
Supplement: Supplementary file 1 — Supplementary file1 (DOCX 291 KB) [file 259_2024_6683_MOESM1_ESM.docx]

**MATERIAL AND METHODS**

**Study design and participants**

The PHERGain trial (NCT03161353) [5] recruited (June 2017-April 2019) in 45 European hospitals (Spain, France, Belgium, Germany, the United Kingdom, Italy, and Portugal). Women (≥18 years) with previously untreated, centrally confirmed, HER2+, stage I-IIIA, invasive, operable BC (≥1.5 cm tumor size by magnetic resonance imagining [MRI] or ultrasound) with ≥1 breast lesion evaluable by FDG-PET (SUVmax ≥1.5 x SUVmean liver + 2 standard deviation [SD]) were included.

A total of 356 patients were randomly assigned (1:4) to either CTX (six cycles of docetaxel-carboplatin) plus concurrent trastuzumab and pertuzumab (group A) or trastuzumab and pertuzumab (group B). HR-positive patients in group B also received letrozole (2.5 mg/day orally) if they were postmenopausal or tamoxifen (20 mg/day orally) if they were premenopausal or perimenopausal. Randomization was stratified by HR status.

Patients allocated to group A received the scheduled treatment regardless of the on treatment FDG-PET results. However, the treatment of group B patients varied according to response after two cycles of treatment: FDG-PET responders continued the same treatment for six more cycles (+/- endocrine therapy) and FDG-PET-non-responders were switched to neoadjuvant CTX (same schedule than group A). Surgery was done 2–6 weeks after the last dose of study treatment. Adjuvant trastuzumab and pertuzumab was continued to complete 1 year of treatment in all patients from both groups (responder and non-responder patients), along with adjuvant endocrine therapy and radiotherapy as per HR status and institutional practices, respectively. In addition, FDG-PET responders in group B who did not achieve a pCR received an additional six cycles of adjuvant CTX plus concurrent trastuzumab and pertuzumab, then four cycles of trastuzumab and pertuzumab.

The study protocol and supporting documents were approved by the institutional review board at each site. All patients provided written informed consent prior to their participation (including a non-specific clause for use of data for biomarker research). This study was conducted in accordance with ethical principles, consistent with the Declaration of Helsinki and International Council of Harmonization/Good Clinical Practice, as well as all applicable regulatory requirements.

**FDG-PET evaluation**

Whole body FDG-PET scans were performed, at baseline (≤ 7 days before treatment initiation) and after two cycles of study treatment, following procedures of the European Association of Nuclear Medicine (EANM) through their EANM Research Limited (EARL) subsidiary.

Standardized Uptake Value (SUV) was calculated as the ratio of tissue radioactivity concentration to the administered dose, divided by bodyweight. SUVmax was defined as the highest pixel SUV within a tumor. See the article of Pérez-García *et al*. [5] for FDG-PET scan procedures.

All the imaging centres were accredited [^18^F]FDG-PET centres of excellence and fulfilled the requirements indicated in the EARL imaging guidelines. The study protocol allowed a maximum of 7 days between baseline [^18^F]FDG-PET imaging and treatment initiation. All [^18^F]FDG-PET scans needed to be done on the same approved device of the imaging centre and identical acquisition and reconstruction settings. Central quality assurance and image analysis, which were done by two independent reviewers, were masked to both the assigned treatment group and clinical outcome data.

At baseline, lesions were grouped according to their location (breast or lymph nodes) and were defined as PET[+] target lesions if they met the following criteria: (1) SUVmax ≥1.5 x SUVmean liver + 2 SD, and (2) longest diameter ≥1.5 cm by MRI or ultrasound. Patients excluded for this reason were considered as PET criteria [-]. For each target lesion, metabolic response was calculated as the percentage decrease from baseline using this formula: (SUVbaseline–SUVresponse)/SUVbaseline. Evaluation of response based on PET images was in accordance with adapted European Organization for the Research and Treatment of Cancer (EORTC) criteria [5].

The threshold for the FDG-PET scan was selected according to the preplanned secondary analysis of the NeoALTTO trial [23], identifying 40% reduction of SUVmax as the most suitable threshold with a satisfactory predictive positive value for pCR and allowing for a realistic trial sample size.

**Stromal tumor-infiltrating lymphocytes (sTILs) analysis**

TILs were reported for the stromal compartment. They were evaluated at baseline on whole slides according to internationally established guidelines, by determining the relative proportion (percentage) of stromal area to tumor area from the pathology slide of a given tumor region.

Centrally performed sTILs were grouped into three categories according to this percentage: low (< 30%), intermediate (30% to < 75%), and high (≥ 75%), based on the high concordance reached between pathologists for the 30% and 75% cutoffs [24].

**PAM50 intrinsic subtyping and risk of recurrence (ROR) scores**

Hematoxylin and eosin-stained formalin-fixed paraffin-embedded (FFPE) breast tissue section (3 µm) was used to confirm the presence of invasive tumor cells and determine the tumor. Ribonucleic acid (RNA) was extracted (RNeasy FFPE Kit, Qiagen, Valencia, CA) from FFPE slides (1-10 µm) of each tumor specimen, performing tumor area microdissection (when needed) to avoid normal breast tissue contamination.

Approximately ≥150 ng of total RNA was used to measure the expression of 50 BC-related genes plus five housekeeping genes (nCounter platform; NanoString Technologies, Seattle WA). Data were Log2 transformed and normalized using the housekeeping genes. Intrinsic subtyping (luminal A, luminal B, HER2-enriched, basal-like, and normal-like) was performed according to the research-based PAM50 intrinsic subtype predictor as previously described [25]. ROR score based on subtype (ROR-S) and subtype and proliferation (ROR-P) were also calculated.

**Vantage 3D^TM^ Cancer Metabolism Panel**

The nCounter^®^ Vantage 3D™ Cancer Metabolism Panel (NanoString Technologies, Seattle, WA, USA), which contains 192 genes related to cancer metabolism, was used for evaluating cancer metabolism. Total RNA (250 ng) was hybridized with probe pools, hybridization buffer, TagSet and incubated at 67 °C for 20 hours. Hybridization, immobilization and purification were performed using the automated nCounter PrepStation (NanoString Technologies, Seattle, WA, USA). Fluorescent barcodes were scanned by nCounter® Digital Analyzer (NanoString Technologies, Seattle, WA, USA), following a high-sensitivity mode (considering 555 felds of view; FOVs) to capture all gene counts. Raw data were collected and pre-processed by nSolver™. Analysis Software version 4.0 (NanoString Technologies, Seattle, WA, USA). Standardized quality control (QC), including imaging QC, binding density, limit of detection QC, positive, and negative controls QC, was conducted for all samples. Only samples fulfilling all QC were eligible for data analysis.

**Objectives**

The objectives of this substudy were (1) to evaluate the association between SUVmax value and FDG-PET status at baseline and clinicopathological features in all screened patients with a tumor size > 1.5 cm by MRI, and (2) to analyze differences in sTILs and gene expression using PAM50 (intrinsic subtyping and ROR scores) and Vantage 3D^TM^ Cancer Metabolism Panel in a matched cohort of excluded and enrolled patients based on the FDG-PET inclusion criteria.

**Statistical analysis**

In all screened patients of PHERGain trial with a tumor size > 1.5 cm by MRI, unadjusted and adjusted analyses based on logistic regression models with Wald test were performed to assess the relationship between SUVmax value at baseline and FDG-PET status ([-]/[+]) with clinicopathological characteristics (tumor size, lymph node involvement, HR status, histological subtype, HER2 protein expression levels by immunohistochemistry [IHC], Ki67 proliferation index, and histological grade).

We randomly selected 21 PET criteria [-] patients with SUVmax levels lower than 2.5 among 75 PET criteria [-] patients screened. We matched them with 21 patients with SUVmax levels equal or higher than 2.5 (PET[+] patients) based on the patient characteristics that turned out to be more associated with FDG-PET status in previous adjusted analysis (tumor size, nodal involvement, and histological grade).

To match each case to a control, we used logistic regression models to calculate propensity scores estimating each patient's probability. The matching method was based on logit distance and the nearest criterion. It was conducted with the library MatchIt from R software. The comparisons between PET criteria [-] and PET[+] paired samples were conducted with Wilcoxon and McNemar’s tests for continuous and categorical outcomes, respectively.

For all statistical analysis, a p-value of less than 0.05 was considered statistically significant. Multiple testing issues with gene expression were controlled with false discovery rate (FDR) using a threshold of q-value<5%.

**REFERENCES**

5. Pérez-García JM, Gebhart G, Ruiz Borrego M, et al (2021) Chemotherapy de-escalation using an 18F-FDG-PET-based pathological response-adapted strategy in patients with HER2-positive early breast cancer (PHERGain): a multicentre, randomised, open-label, non-comparative, phase 2 trial. Lancet Oncol 22:858–871. https://doi.org/10.1016/S1470-2045(21)00122-4

23. de Azambuja E, Holmes AP, Piccart-Gebhart M, et al (2014) Lapatinib with trastuzumab for HER2-positive early breast cancer (NeoALTTO): survival outcomes of a randomised, open-label, multicentre, phase 3 trial and their association with pathological complete response. Lancet Oncol 15:1137–1146. <https://doi.org/10.1016/S1470-2045(14)70320-1>

24. Kos Z, Roblin E, Kim RS, et al (2020) Pitfalls in assessing stromal tumor infiltrating lymphocytes (sTILs) in breast cancer. NPJ Breast Cancer 6:17. <https://doi.org/10.1038/s41523-020-0156-0>

25. Llombart-Cussac A, Cortés J, Paré L, et al (2017) HER2-enriched subtype as a predictor of pathological complete response following trastuzumab and lapatinib without chemotherapy in early-stage HER2-positive breast cancer (PAMELA): an open-label, single-group, multicentre, phase 2 trial. Lancet Oncol 18:545–554. <https://doi.org/10.1016/S1470-2045(17)30021-9>

**SUPPLEMENTARY FIGURE**


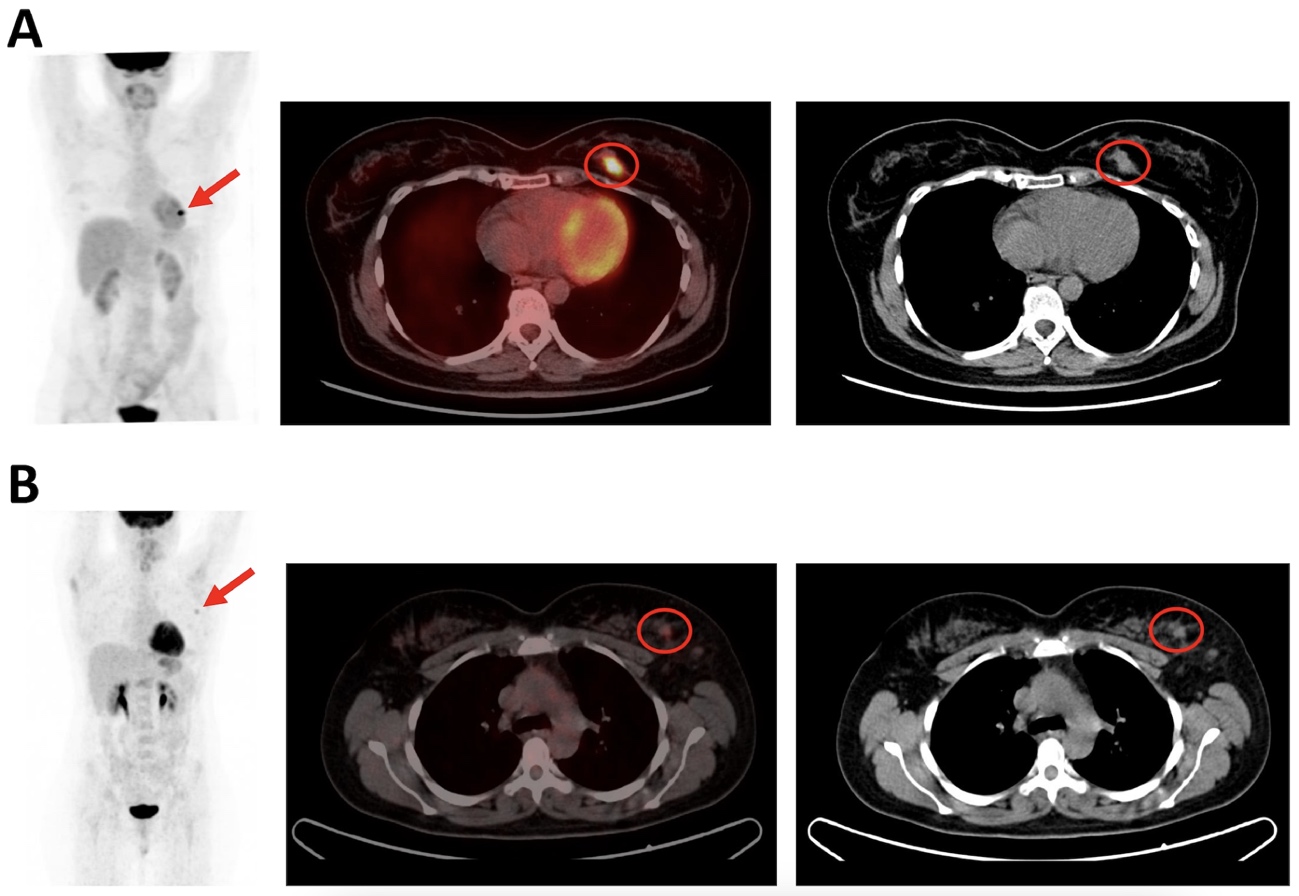


**Supplementary Figure 1.** Representative differences between a PET[+] patient (A) and a PET[-] patient (B) obtained by FDG-PET scans.
